# Puzzling Out the Genetic Architecture of Endometriosis: Whole-Exome Sequencing and Novel Candidate Gene Identification in a Deeply Clinically Characterised Cohort

**DOI:** 10.3390/biomedicines11082122

**Published:** 2023-07-27

**Authors:** Aurora Santin, Beatrice Spedicati, Anna Morgan, Stefania Lenarduzzi, Paola Tesolin, Giuseppe Giovanni Nardone, Daniela Mazzà, Giovanni Di Lorenzo, Federico Romano, Francesca Buonomo, Alessandro Mangogna, Maria Pina Concas, Gabriella Zito, Giuseppe Ricci, Giorgia Girotto

**Affiliations:** 1 Department of Medicine, Surgery and Health Sciences, University of Trieste, 34149 Trieste, Italy; aurora.santin@burlo.trieste.it (A.S.); paola.tesolin@burlo.trieste.it (P.T.); giuseppegiovanni.nardone@burlo.trieste.it (G.G.N.); giuseppe.ricci@burlo.trieste.it (G.R.); giorgia.girotto@burlo.trieste.it (G.G.); 2 Institute for Maternal and Child Health, I.R.C.C.S. “Burlo Garofolo”, 34137 Trieste, Italy; anna.morgan@burlo.trieste.it (A.M.); stefania.lenarduzzi@burlo.trieste.it (S.L.); daniela.mazza@burlo.trieste.it (D.M.); giovanni.dilorenzo@burlo.trieste.it (G.D.L.); federico.romano@burlo.trieste.it (F.R.); francesca.buonomo@burlo.trieste.it (F.B.); alessandro.mangogna@burlo.trieste.it (A.M.); mariapina.concas@burlo.trieste.it (M.P.C.); gabriella.zito@burlo.trieste.it (G.Z.)

**Keywords:** endometriosis, whole-exome sequencing, rare variants, candidate genes, deep clinical evaluation, genotype–phenotype correlations

## Abstract

Endometriosis (EM) is a common multifactorial gynaecological disorder. Although Genome-Wide Association Studies have largely been employed, the current knowledge of the genetic mechanisms underlying EM is far from complete, and other approaches are needed. To this purpose, whole-exome sequencing (WES) was performed on a deeply characterised cohort of 80 EM patients aimed at the identification of rare and damaging variants within 46 EM-associated genes and novel candidates. WES analysis detected 63 rare, predicted, and damaging heterozygous variants within 24 genes in 63% of the EM patients. In particular, (1) a total of 43% of patients carried variants within 13 recurrent genes (*FCRL3*, *LAMA5*, *SYNE1*, *SYNE2*, *GREB1*, *MAP3K4*, *C3*, *MMP3*, *MMP9*, *TYK2*, *VEGFA*, *VEZT*, *RHOJ*); (2) a total of 8.8% carried private variants within eight genes (*KAZN*, *IL18*, *WT1*, *CYP19A1*, *IL1A*, *IL2RB*, *LILRB2*, *ZNF366*); (3) a total of 24% carried variants within three novel candidates (*ABCA13*, *NEB*, *CSMD1*). Finally, to deepen the polygenic architecture of EM, a comprehensive evaluation of the analysed genes was performed, revealing a higher burden (*p* < 0.05) of genes harbouring rare and damaging variants in the EM patients than in the controls. These results highlight new insights into EM genetics, allowing for the definition of novel genotype–phenotype correlations, thereby contributing, in a long-term perspective, to the development of personalised care for EM patients.

## 1. Introduction

Endometriosis (EM) is a chronic oestrogen-dependent disease characterised by the ectopic presence of active endometrium outside the uterine cavity, as in the myometrium (i.e., adenomyosis), ovaries, uterosacral ligaments, bladder, and pelvic peritoneum, and even out of the pelvis as well [1]. It is a common gynaecological disorder, affecting approximately 10–15% of women of reproductive age [1,2]; however, considering that some affected patients may remain paucisymptomatic with advanced EM and, conversely, even extremely distressing symptoms may be overlooked, the disease is probably underdiagnosed, and the current estimates do not reflect the true prevalence of the disorder [3]. The clinical presentation of EM can be subtle [1]. Additionally, affected women often experience infertility [4].

To date, there are several hypotheses to explain EM aetiology [5]. The most widely accepted is the “retrograde menstruation” theory, recently updated with the “stem cell” one [6]. However, it still does not explain (i) why the retrograde menstruation, which happens in the majority of women, leads to EM only in a small percentage of them, and (ii) the occurrence of EM in females with Rokitansky syndrome [7] and in males [8]. Therefore, the biological and molecular pathways involved in the etiopathogenesis of this disorder are still unclear.

EM is recognised as a multifactorial disorder, in which both genetic and environmental factors play significant roles [9]. Concerning the environmental factors, pollution exposure and diet appear to be the main ones involved. For instance, dioxin (2,3,7,8-tetrachlorodibenzo-p-dioxin (TCDD)) has been widely implicated in EM pathogenesis [10,11], while regarding diet, phytoestrogens and saturated fats have been linked with this disease [12].

Aside from the environmental factors, several studies have highlighted the contribution of genetics to the aetiology of this complex disease. The first formal genetic study on EM was conducted in 1971 by Simpson and colleagues, who demonstrated the familial clustering of this disorder [13]. Since then, several additional family studies have been performed, showing a higher concordance for monozygotic tweens than dizygotic couples, and wide population-based studies have estimated that the heritability of EM is approximately 50%, suggesting that it may follow a polygenic inheritance pattern [14]. In recent years, Genome-Wide Association Studies (GWASs) have proven to be an effective tool to identify EM-associated genes [15,16]; many of them appear to be involved in sex-steroid-hormone signalling, *WNT* signalling, cell adhesion and migration, cell growth, and inflammation-related pathways. Although GWASs have been extremely useful in identifying many genes potentially involved in the etiopathogenesis of EM, their findings are able to explain less than 5% of the phenotypic variance; additionally, the exact causative link between the identification of a susceptibility locus and the underlying molecular pathways that leads to disease development remains, in many cases, unclear [16]. In this light, new approaches are needed to fill the current knowledge gap regarding EM pathophysiology to allow for the identification of novel molecular targets that can be implemented as diagnostic, prognostic, and therapeutic biomarkers.

To this purpose, in this study, we performed whole-exome sequencing (WES) analysis on a highly selected cohort of 80 deeply characterised patients with the final goals of identifying rare variants in 46 known EM-associated genes and discovering new potentially causative ones. Furthermore, in order to deepen the complex polygenic nature of EM, a comprehensive evaluation of the analysed genes was performed, testing the hypothesis that the burden of genes harbouring rare and damaging variants is higher in EM patients than in a control cohort.

## 2. Materials and Methods

### 2.1. Ethical Statement

Written informed consent to participate in the study and for the collection of biological samples for research purposes was obtained from all participants. The study was conducted in accordance with the Helsinki Declaration and was approved by the Ethics Committee of the Friuli-Venezia Giulia region (Italy) (Prot. n. 47846 dd. 27.12.2022).

### 2.2. Participants’ Recruitment and Clinical Evaluation

A total of 80 adult women with confirmed surgical or clinical diagnoses of EM were recruited at the I.R.C.C.S. “Burlo Garofolo” Hospital in Trieste (Italy). Confirmation of the EM diagnosis was based on the following: (1) both visual inspection with histological confirmation for the patients that underwent surgery (i.e., laparoscopy or laparotomy); (2) imaging techniques performed by expert operators, for the remaining subjects, such as transvaginal or transrectal and transabdominal ultrasound according to the International Deep Endometriosis Analysis (IDEA) group consensus [17], and/or magnetic resonance imaging (MRI) according to the European Society of Urogenital Radiology (ESUR) guidelines [18]. The EM severity was staged considering the revised American Society for Reproductive Medicine (rASRM) classification [19].

At enrolment, all patients underwent a deep clinical evaluation. In particular, detailed information regarding demographic data (i.e., age, anthropometric measurements), past and familial medical history, and gynaecological anamnesis (i.e., age of menarche, EM diagnosis, number of pregnancies, infertility diagnosis) were collected. Furthermore, a careful evaluation of the most common EM-associated symptoms (i.e., ovulation, pre-menstrual and post-menstrual pain, dysmenorrhea, dyspareunia, dyschezia, dysuria) before medical therapy or surgery was performed for each patient.

Specifically, the presence of the abovementioned pain was registered as a dichotomous variable (“yes”/“no”), and the intensity of pain was rated with a 0–10-point Numerical Rating Scale (NRS) (i.e., 0 represents “no pain at all” and 10 represents “the worst imaginable pain”) [20].

### 2.3. DNA Extraction and Quality Control

For each patient, a peripheral whole-blood sample was collected for genomic DNA extraction. The protocol for genomic DNA extraction was performed as already described in Spedicati et al. [21].

### 2.4. Whole-Exome Sequencing (WES)

WES was carried out on an Illumina NextSeq 550 instrument (Illumina Inc., San Diego, CA, USA) with the Twist Exome 2.0 plus Comprehensive Exome Spike-in kit (Twist Bioscience, South San Francisco, CA, USA), according to the manufacturer’s protocol. The WES protocol and secondary and tertiary analyses were carried out as already reported in Spedicati et al. [21].

### 2.5. WES Data Analysis and Variant Selection

Two different phases of WES data analysis were performed. Firstly, a candidate gene approach was carried out, focusing the WES data analysis on a list of EM-associated genes; secondly, an unbiased approach was conducted to detect variants within novel candidate genes. The complete list of the analysed genes is reported in Table S1.

As regards the candidate gene approach, a list of 46 genes was created based on a literature review, according to the following criteria: (i) each gene had to be described in association with EM in at least two published papers; (ii) only the most recent papers (i.e., published between 2011 and 2023) were considered.

To perform variant selection within the analysed genes and novel candidates, the following criteria were applied:

1. Variants with a quality score < 20, Variant Allele Frequency < 30, or called in off-target regions were excluded;
2. A Minor Allele Frequency (MAF) cut-off of 0.1% was considered. The variant frequency was verified both in NCBI dbSNP (https://www.ncbi.nlm.nih.gov/snp/, accessed on 30 April 2023) and gnomAD (https://gnomad.broadinstitute.org/, accessed on 30 April 2023);
3. The effect of the genetic variants was evaluated with *in silico* prediction tools, such as PolyPhen-2 (tolerated for scores < 0.5, damaging for scores ≥ 0.5) [22], SIFT [23], PaPI (tolerated for scores ≤ 0.5, damaging for scores > 0.5) [24], DANN (tolerated for scores ≤ 0.9, damaging for scores > 0.9) [25], the dbscSNV score (tolerated for scores ≤ 0.9, damaging for scores > 0.9) [26], and SpliceAI (tolerated for scores < 0.5, probably damaging for scores ranging from 0.5 to 0.8, damaging for scores ≥ 0.8) [27];
4. SNVs leading to synonymous aminoacidic substitutions not predicted as damaging, not affecting splicing, or highly conserved residues were excluded.

To interpret the effect of the selected variants, the genetic intolerance profile for the protein domains was analysed with the MetaDome web server [28].

Finally, the correlation between each patient’s variants and clinical phenotype was examined, evaluating the related literature, in order to identify possible relevant genotype–phenotype correlations. All selected variants were confirmed via Sanger sequencing.

### 2.6. Control Cohort

One hundred and five healthy women recruited during routine gynaecological visits to I.R.C.C.S. “Burlo Garofolo” (Trieste, Italy) were included in this study as a control cohort. This control group was carefully selected according to the following criteria: (1) age over 18 years, (2) absence of clinical and/or surgical diagnosis of EM, and (3) no reports of infertility issues. WES data of these subjects were already available as an in-house database and were analysed following the criteria described in this paragraph.

### 2.7. Statistical Analysis

The “burden of genes” was calculated for each individual of the EM and control cohorts. In particular, for each participant, the burden of genes was defined as the total number of genes, considering variants within the 46 selected genes and the novel candidates identified, in which at least one rare and damaging variant was identified after the WES analysis and variant selection. A Wilcoxon two-sample rank test was performed to compare the burden of genes’ distribution between the cases and controls. The statistical significance was set to a *p*-value < 0.05. The analysis was performed with R version 4.1.2 (R Foundation for Statistical Computing, Vienna, Austria).

**Figure 1:**
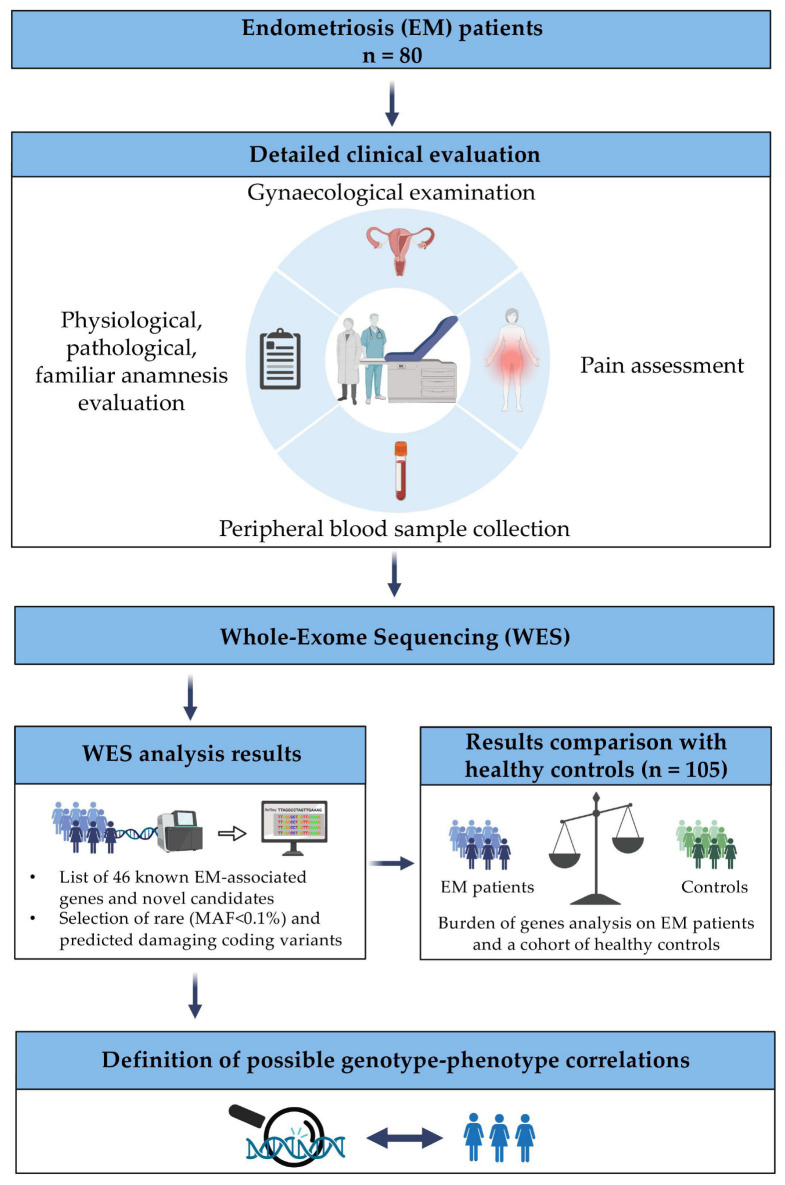
Schematic representation of the study workflow. A total of 80 adult patients with confirmed EM diagnoses were enrolled at I.R.C.C.S. “Burlo Garofolo” Hospital in Trieste (Italy). All the patients underwent a deep clinical evaluation, during which detailed information was collected regarding demographic data, past and familial medical history, gynaecological anamnesis, and EM-associated symptoms. Further, for each patient, a peripheral blood withdrawal was collected for WES analysis. The WES results were then compared with the WES data of the 105-healthy-women cohort. Finally, the correlation between the identified variants and the clinical phenotype of each patient was examined. Created with BioRender.com (accessed on 21 May 2023).

The complete workflow of this study is reported in Figure 1.

## 3. Results

### 3.1. Demographic Data and Clinical Features of EM Patients

A cohort of 80 EM patients aged between 20 and 57 years were enrolled at I.R.C.C.S. “Burlo Garofolo” Hospital (Trieste, Italy). In particular, 59/80 (74%) patients were diagnosed with EM stage III–IV, and 95% of them underwent surgery. Furthermore, 53% of the EM patients had full-term pregnancies, and 21% received infertility diagnoses. Forty-five percent of patients were currently undergoing a medical therapy (i.e., progestins, combined oestrogen–progestins, or hormone-releasing intrauterine devices), while only 16% took only anti-inflammatory drugs to control EM-associated symptoms. Notably, only 5.8% of patients suffered from dysuria, while most of them (64%) reported dysmenorrhea, with a mean pain intensity rating of 6.8 on the NRS. Complete patient demographic data and clinical features are summarised in Table 1.

### 3.2. WES Analysis and Results Classification

The WES analysis was performed in two steps, firstly focusing on the selected list of 46 EM-associated genes, and then on the novel candidate discovery.

Overall, this strategy allowed for the identification of 63 rare (MAF < 0.1%), predicted, and damaging variants within 21/46 genes and 3 novel candidates (*ABCA13*, *NEB*, and *CSMD1*) in 50/80 (63%) EM patients. All variants were detected in the heterozygous state.

**Figure 2:**
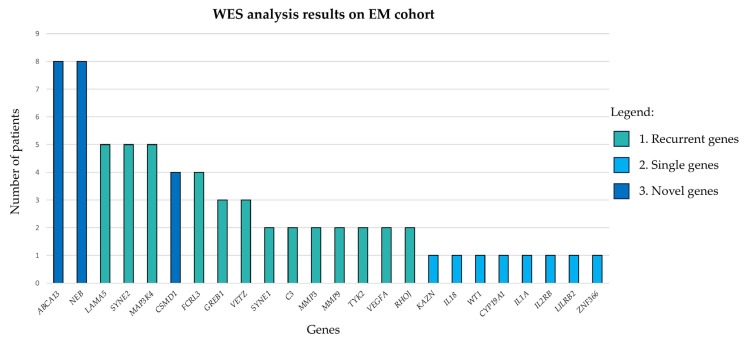
WES analysis results of EM cohort. The bar plot shows, for each gene (*x*-axis), the number of EM patients carrying rare, predicted, and damaging variants (*y*-axis) in descending order. Results are represented in a colour code according to WES result classification reported in Section 3.2: (1) the recurrent genes are reported in the blue colour, (2) the single genes are reported in turquoise, and (3) the novel candidate genes identified are reported in light blue.

Complete WES results are reported in Table S2 and graphically represented in Figure 2.

In particular, the WES analysis results revealed the following:

1. A total of 34/80 (43%) patients carried different rare, predicted, and damaging variants within 13 recurrent genes (*FCRL3*, *LAMA5*, *SYNE1*, *SYNE2*, *GREB1*, *MAP3K4*, *C3*, *MMP3*, *MMP9*, *TYK2*, *VEGFA*, *VEZT*, *RHOJ*);
2. A total of 7/80 (8.8%) patients (i.e., patients 3, 9, 19, 50, 67, 14, 19, 13) carried different private, rare, predicted, and damaging variants within eight single genes (*KAZN*, *IL18*, *WT1*, *CYP19A1*, *IL1A*, *IL2RB*, *LILRB2*, *ZNF366*);
3. A total of 19/80 (24%) patients carried different rare, predicted, and damaging variants within three novel candidate genes (*ABCA13*, *NEB*, *CSMD1*).

Further, the WES analysis detected the following: (i) the *FCRL3* (NM_052939.4):c.958T>A variant segregated in patient 34 and her sister (patient 35); (ii) the *MAP3K4* (NM_005922.4):c.3590_3598dupCTGCTGCTG variant detected in patient 28 segregated in two other members of her family (patients 63 and 64 (respectively, her mother and aunt)), both diagnosed with EM.

### 3.3. Rare Variants within Recurrent Genes

Regarding the recurrent genes mentioned above (Table 2), the most compelling results regard the *FCRL3*, *LAMA5*, *SYNE1*, and *SYNE2* genes. Four out of eighty patients (5.0%) carried different predicted and damaging variants within the *FCRL3* gene. To note, patient 2 had an infertility diagnosis, and patients 34 and 35, two sisters, reported a family history of infertility.

In 5/80 (6.3%) of the EM patients, the WES analysis allowed for the identification of different novel, predicted, and highly impacting missense variants within the *LAMA5* gene; all these selected variants belong to domains of the encoded protein predicted as intolerant to missense variation. Three out of five patients (patients 5, 12, and 23) carrying *LAMA5* variants were diagnosed with EM stage III–IV.

Moreover, in 7/80 (8.8%) patients, different rare, predicted, and damaging variants within two genes belonging to the same family, *SYNE1* and *SYNE2*, were identified. Notably, six out of seven of the patients (patients 16, 23, 26, 40, 45, and 47) carrying variants within these two genes shared a common clinical feature: a severe, EM-associated, and painful symptomatology, often poorly controlled via antalgic and medical therapy.

### 3.4. Rare, Private Variants within Specific Genes in Single Patients

In 7/80 (8.8%) EM patients, the data analysis identified rare, private, predicted, and damaging variants within single genes (Table 3) that, according to the literature, may have a role in regulating the inflammatory response and infertility mechanisms underlying EM pathogenesis. The most enthralling results concern the *IL18*, *KAZN*, and *WT1* genes.

In details, patient 3, a 30-year-old woman with EM stage IV, carried a predicted and damaging missense variant within the *IL18* gene. The genetic-intolerance-profile analysis with MetaDome revealed that this variant belongs to a protein domain predicted as highly intolerant to missense variation.

Patient 9, a 49-year-old woman with EM stage IV, showed a predicted and damaging missense variant within the *KAZN* gene. A careful evaluation of patient 9’s clinical history revealed that this patient had three pregnancies that all ended with spontaneous miscarriages.

In patient 19, a predicted and damaging missense variant within the *WT1* gene was detected. Of note, patient 19 had been diagnosed with infertility and reported severe dysmenorrhea, rated eight on the NRS.

### 3.5. Identification of Novel Candidate Genes

The WES data analysis carried out with an unbiased approach revealed that 19/80 (24%) EM patients carried rare, predicted, and damaging variants in three novel candidate genes (*ABCA13*, *NEB*, *CSMD1*) (Table 4). In particular, 8/80 (10%) of the EM patients carried different predicted and damaging variants within the *ABCA13* gene, 8 other patients within the *NEB* gene, and the remaining 4 within the *CSMD1* gene.

### 3.6. Burden of Genes Analysis

From a genetic perspective, EM is a polygenic disorder determined by the combined effect of multiple genes. In order to evaluate whether a higher number of genes harbouring rare and damaging variants was present in EM patients than in healthy controls, the “burden of genes” was calculated. Specifically, for each individual of the EM and control cohorts, the burden of genes was defined as the total number of genes in which at least one rare and damaging variant was identified (Table S3). The burden of genes’ distribution ranged [0–3] both in the cases and controls. The medians and interquartile ranges were 1.0 (0.0–1.0) in the EM patients and 0.0 (0.0–1.0) in the controls. A Wilcoxon two-sample rank test determined that the burden of genes was statistically significantly higher *(p* = 0.02) in the EM cohort than in the controls.

## 4. Discussion

EM is a chronic, inflammatory, multifactorial disease with a high prevalence in the general population. To date, it is one of the most underdiagnosed and undertreated disorders, with a mean of 8–12 years between the beginning of symptoms and a definitive diagnosis [29]. Furthermore, EM is characterised by a heavy social impact, as it has detrimental effects on women’s quality of life, fertility, and social relationships [30]. The current unavailability of rapid and minimally invasive diagnostic tools poses a complexity for clinicians in the diagnostic process of and therapeutic planning for EM. Therefore, there is an urgent clinical need to detangle the complex genetic and molecular mechanisms underlying this disease’s etiopathogenesis.

Several GWASs have been carried out so far to pinpoint novel EM-associated genes. However, although GWASs are a successful strategy to identify genetic variants underlying multifactorial disorders, they present some limitations. Firstly, GWASs select variants that are associated with the disease of interest, rather than a causal mechanism. Secondly, GWASs can detect only relatively common variants widespread in the population. Thirdly, GWASs require a precise phenotypical characterisation to obtain solid and reliable results, which may be less accurate in large-sample cohorts. For this reason, other genetic approaches are needed.

In this light, this study took advantage of a combined approach of a detailed clinical characterisation and WES analysis to deepen, for the first time in the literature, the effect of rare variants within a list of highly selected EM-associated genes and novel candidates. This strategy allowed us to perform accurate genotype–phenotype comparisons, unveiling interesting insights into EM’s underlying pathological mechanisms.

The WES analysis identified 63 predicted and damaging variants within 21 genes and 3 novel candidates. All these genes belong to several molecular pathways, such as the regulation of the immune response, cellular proliferation and migration, and oestrogen metabolism, all reported to be involved in EM pathogenesis [31]. This is a particularly relevant result considering that thorough research of the literature revealed that only a few causative variants and genes are currently described in relation to EM [32].

The most compelling results regard the *FCRL3*, *LAMA5*, *SYNE1*, and *SYNE2* genes, in which rare, predicted, and damaging variants were detected in more than one patient, and a consistent genotype–phenotype correlation was identified.

Concerning *FCRL3*, a gene encoding a member of the immunoglobulin receptor family, rare and damaging variants were identified in 4/80 (5.0%) EM patients. Three out of four of these patients reported a diagnosis and/or family history of infertility. According to the literature, this gene has already been linked with an increased risk of EM-associated infertility, irrespective of the disease stage [33,34]. To date, the cause-and-effect relation underlying EM and infertility is still poorly elucidated, and several genes regulating inflammation and angiogenesis are currently being explored as potential etiologic factors; among them, *FCRL3* is a promising candidate. Indeed, *FCRL3*, besides B cells, is also expressed in natural killer (NK) cells and regulatory T cells (Treg), key modulators of the specific immune response against ectopic endometrial lesions and involved in fertility-mechanism modulation [35]. Previous studies have shown that the increased levels of *FCRL3*-positive Tregs detected in EM patients could be responsible for a reduced immune response that could enable the implantation of endometrial cells and infertility onset [36]. In this light, an in-depth characterisation of the *FCRL3* gene’s role in relation to infertility mechanisms could pave the way, in the future, for novel strategies for the better early clinical management of patients with infertility issues.

Regarding *LAMA5*, this gene encodes the alpha-5 Laminin protein, which has been associated with EM stage III–IV and EM-related infertility [37,38]. In particular, high levels of LAMA5 were detected in the eutopic endometria of EM-stage-III–IV patients during the menstrual proliferative phase, and an association of a *LAMA5* SNP (rs2427284) with EM stage III–IV has been demonstrated [37,38]. In accordance with these findings, in our cohort, three out of five patients carrying variants within this gene were diagnosed with EM stage III–IV, giving relevance to the previously described relation. Further, all five identified *LAMA5* missense variants belong to protein domains predicted as intolerant to missense variation. Therefore, it can be hypothesised that these variants impact on the LAMA5 structure and biological function, thereby promoting the adhesion of endometrial cells in ectopic sites. From this perspective, further studies are needed to unveil the relationship between *LAMA5* variants and EM stage III–IV. This could be relevant to gain a deeper understanding of EM progression mechanisms, and to evaluate the potential predictive and prognostic values of rare and damaging variants within this gene.

Finally, as regards *SYNE1* and *SYNE2*, another relevant genotype–phenotype correlation was identified. *SYNE1* and *SYNE2* are two genes belonging to the same family, encoding, respectively, the Nesprin1 and Nesprin2 proteins, two structural proteins that share a common function of anchoring the nuclear envelope to the actin cytoskeleton. Notably, six out of seven EM patients carrying damaging variants within these genes reported a severe EM-associated symptomatology. Indeed, *SYNE1* has been recently associated with the most common EM-associated pain symptoms (e.g., dysmenorrhea, dyspareunia, severe dyspareunia, and acyclic pelvic pain) and menstrual migraine [16]. To note, this gene belongs to the same genomic locus of other genes (i.e., *ESR1* and *CCDC170*) involved in oestrogen-hormone signalling, a key player in the regulation of the growth of endometriotic lesions, and in the modulation of pain perception [15]. Concerning *SYNE2*, its expression was found to be dysregulated in the endometria of EM patients [39] and, currently, there are no available data associating it with EM-related pain. However, considering that this gene belongs to the same gene family as *SYNE1*, it could be speculated that *SYNE2* could play a similar role in EM pathogenesis; hence, further functional characterisations of *SYNE2* variants are needed to elucidate the role of this gene in EM-associated pain perception.

Overall, deepening the role of *SYNE1* and *SYNE2* variants in relation to EM-associated pain could be fundamental to shed light on the entangled mechanism underlying EM symptom severity and variability. This will pave the way for the identification of novel molecular markers to be implemented, in the future, in clinical practice, thereby allowing for the better clinical management of patients carrying *SYNE1/SYNE2* variants, with the activation of personalised treatment plans to improve patients’ symptomatology and quality of life.

Of particular interest also are the *IL18*, *KAZN*, and *WT1* genes, in which private variants in single patients were detected and a genotype–phenotype correlation was defined.

Concerning the *IL18* gene, encoding Interleukin 18, several studies have demonstrated that this gene regulates the immune response in the human endometrium, activating NK cells. A previous study showed that the *IL18* expression levels in the endometria of EM patients are lower compared to healthy women [40], and this is associated with decreased NK-cell activity, thus allowing endometriotic lesions to escape immune elimination [41]. Further, according to the literature, *IL18* also plays a role in female fertility maintenance, regulating uterine receptivity and the embryo-implantation phase [42]. Indeed, previous studies have shown that women with repeated failures of implantation after in vitro fertilisation showed the dysregulation of *IL18* and other cytokines in the endometrium, compared with fertile women [42]. All these findings suggest that *IL18* could be a relevant player not only in EM pathogenesis, but also in EM-related infertility mechanisms, defining it as a promising molecular target to be implemented, in a long-term perspective, as a predictive and diagnostic biomarker.

Moreover, the *KAZN* gene, which encodes a desmosomal protein involved in cell adhesion, cytoskeleton organisation, and embryonic tissue morphogenesis, has also been linked with EM and EM-related infertility [37]. A compelling genotype–phenotype correlation was found in this study, as patient 3, carrying a damaging missense variant within the *KAZN* gene, had three pregnancies, all of which ended with spontaneous miscarriages. According to the literature, variants within the *KAZN* gene have also been associated with infertility and pregnancy- and labour-related complications [43]. All these findings led to the hypothesis that *KAZN* could play a relevant role in fertility- and pregnancy-related mechanisms. In this perspective, the characterisation of damaging variants within this gene could allow for novel genetic insights into EM-related infertility and pregnancy complications, thereby paving the way for the identification of new candidate markers to be implemented in clinical routine for early diagnostic and preventive strategies.

Finally, the *WT1* gene encodes a zinc-finger-containing transcription factor that regulates female fertility [44] and has recently been linked with EM-associated dysmenorrhea [16]. Further, it has been reported that *WT1* is involved in determining an aberrant increase in aromatase expression and oestrogen synthesis in the eutopic and ectopic endometria of EM patients [45]. In this cohort, a reasonable genotype–phenotype correlation was detected, as a damaging missense variant within the *WT1* gene was identified in patient 19, diagnosed with EM, EM-related infertility, and severe dysmenorrhea. Considering the strength of the genotype–phenotype correlation identified, *WT1* could be a promising biomarker; indeed, it could be considered in future clinical practice for the definition of the tailored clinical management of patients carrying variants within this gene aimed at (1) reducing the severity of EM-associated dysmenorrhea and (2) implementing ad hoc preventive strategies for fertility maintenance.

A noteworthy feature of this study is represented by the peculiar enrichment of EM patients carrying variants within three novel candidate genes, *ABCA13*, *NEB*, and *CSMD1*, which were selected considering their potential biological role in relation to EM pathogenesis. In particular, the *ABCA13* gene, encoding a ganglioside transporter [46], could be a novel candidate to deepen the entangled mechanisms underlying pain processing. Indeed, alterations in ganglioside metabolism are related to neuropathic and inflammatory pain [47]. Considering also that variants within the *ABCA13* gene are associated with an increased susceptibility to schizophrenia, bipolar, and major depression disorders [46], and that EM has been associated with depression and a higher incidence of anxiety [30], this gene could be a novel player to also scrutinise these aspects.

The role of *NEB* in EM’s pathogenesis has yet to be clarified. This gene encodes Nebulin, a sarcomere protein that regulates cytoskeletal dynamics [48]. Considering that, in EM pathogenesis, the dynamic remodelling of cytoskeleton components is involved in the migration of endometriotic lesions, *NEB* could be a novel candidate to be considered in this process. In line with this, literature data report that the *NEB* gene is frequently mutated in stage III endometrial cancer [49], and other genes of the Nebulin family, (e.g., *LASP1*, *LASP2*) are involved in cytoskeletal-architecture regulation and focal-adhesion organisation [48].

Finally, *CSMD1* encodes a regulator of the complement system, the biological role in regulating fertility mechanisms and cellular proliferation [50] of which would allow for intriguing insights into EM pathogenesis. Only one GWAS in the literature has reported this gene in relation to EM, but the variant identified did not reach genome-wide significance [51]. Several studies are currently highlighting its role in regulating cellular proliferation, as *CSMD1* inhibition causes increased cellular invasion, motility, and proliferation [52]. Therefore, it can be hypothesised that variants within *CSMD1* could impact its biological function, thereby promoting the migration and proliferation of endometrial cells in ectopic sites. However, further in vitro and/or in vivo studies are necessary to characterise the role of *CSMD1* in EM onset.

Finally, in order to deepen the complex genetic architecture of EM, a comprehensive evaluation of the analysed genes was performed in this study. Specifically, a statistically significant (*p* < 0.05) higher burden of the analysed genes harbouring at least one rare and damaging variant was detected in EM patients in comparison to healthy individuals. This result, considering the polygenic nature of EM, could be an intriguing breakthrough into the entangled EM genetic architecture, highlighting the potentially relevant involvement of these genes in this disease’s aetiology.

## 5. Conclusions

In conclusion, this study relied on a comprehensive, deep clinical evaluation and a WES analysis approach that, for the first time in the literature, allowed for the identification of novel and accurate genotype–phenotype correlations in an EM cohort, with a potential translational value into clinical practice. In-depth characterisation of the variants identified will be needed to confirm their biological relevance in EM onset and progression, thereby laying the foundation, in a long-term perspective, for the definition of novel and tailored treatment strategies for the better clinical management of EM patients.

Moreover, three promising candidate genes (i.e., *ABCA13*, *NEB*, and *CSMD1*) were detected, allowing for new genetic insights into EM aetiopathogenesis. Replication studies in independent cohorts and functional experiments will be needed to further characterise the roles of these genes and variants to gain a deeper understanding of their effects in relation to EM.

In 30/80 (37.5%) of the analysed patients, no damaging variants within the selected genes were detected; this suggests that other players might be involved, underlying how the genetic landscape underlying EM is extremely complex.

In this light, the combined approach of an accurate clinical characterisation and a careful analysis of genetic data toward new candidates could be a successful strategy to identify novel molecular markers that, in the future, will make a substantial contribution to improving diagnostic and treatment strategies, thereby paving the way for personalised clinical management.

## Figures and Tables

**Table 1:** Demographic and clinical data of endometriosis (EM) patients. The table reports the main demographic and clinical data of EM patients described as mean and standard deviation (Mean ± sd) or number (N) and percentage (%) of patients. rASRM classification: EM diagnosis staged according to the revised American Society for Reproductive Medicine classification. VTP: voluntary termination of pregnancy. MAP: medically assisted procreation. EM medical therapy: number (N) and percentage (%) of patients that underwent a medical therapy for EM at least once. Ongoing medical therapy: number (N) and percentage (%) of patients currently undergoing a medical therapy for EM. Anti-inflammatory drugs only: number (N) and percentage (%) of patients that followed a therapy only based on anti-inflammatory drugs. Pain evaluation: assessment of the presence and intensity of EM-associated pain before medical therapy or surgery, or at baseline. The presence of pain symptoms was registered as a dichotomous variable (“yes”/“no”), and the intensity of pain was rated with the Numerical Rating Scale (NRS). * Available data for 68/80 patients; ** available data for 31/36 patients; *** available data for 69/80 patients.

Demographic and Clinical Data (n = 80)	Mean ± sd or N (%)
Age (years)	37.6 ± 9.2
Body-mass index (kg/m^2^)	22.9 ± 4.0
Age at menarche (years)	12.1 ± 1.5
**EM diagnosis**	
Surgery and histopathological exam	73 (91.3%)
Transvaginal and transrectal ultrasound and/or magnetic resonance imaging (MRI)	7 (8.75%)
**rASRM classification**	
Stage I	8 (10.0%)
Surgical confirmation	4 (5.00%)
Stage II	13 (16.3%)
Surgical confirmation	13 (16.3%)
Stage III–IV	59 (73.8%)
Surgical confirmation	56 (70.0%)
**Gynaecological data**	
Pregnancies	42 (52.5%)
Parity	38 (47.5%)
Term Labour	38 (47.5%)
Abortion	14 (17.5%)
VTP	4 (5.00%)
MAP	2 (2.50%)
Infertility diagnosis *	14 (20.6%)
**EM Medical therapy**	50 (62.5%)
Ongoing medical therapy	36 (45.0%)
Progestins **	16 (51.6%)
Combined oestrogen–progestins **	11 (35.5%)
Hormone-releasing intrauterine devices (IUDs) **	4 (5.00%)
Anti-inflammatory drugs only **	13 (16.3%)
Pain evaluation and intensity assessment ***	
Ovulation pain	28 (40.6%)
Intensity	6.9 ± 2.1
Pre-menstrual pain	27 (39.1%)
Intensity	6.3 ± 2.4
Post-menstrual pain	14 (20.3%)
Intensity	8.1 ± 2.0
Dysmenorrhea	44 (63.8%)
Intensity	6.8 ± 1.8
Dyspareunia	30 (43.5%)
Intensity	6.0 ± 2.5
Dyschezia	24 (34.8%)
Intensity	5.4 ± 2.0
Dysuria	4 (5.8%)
Intensity	5.0 ± 3.5

**Table 2:** Variants within recurrent genes identified through WES analysis. The table displays the rare, predicted, and damaging variants within common genes identified in more than one patient of the EM cohort. All variants were detected at the heterozygous state. Gene name (isoform), size: name of the gene, isoform, and gene size. HGVS coding, protein: cDNA and protein change variant description according to the Human Genome Variation Society (HGVS) nomenclature guidelines. AF: gnomAD allele frequency. PaPI, PolyPhen, SIFT, DANN, dbscSNV, SpliceAI: variant effect evaluated via *in silico* prediction tools. MetaDome analysis: genetic-tolerance-profile domain of the identified variant. Patient ID: unique identifier of the patient carrying the selected variants. * stop codon. ¶: patients with a degree of kinship. NA: not available. D: damaging. T: tolerated.

Gene Name (Isoform), Size	HGVS Coding, Protein	AF	PaPI	PolyPhen	SIFT	DANN	dbscSNV	SpliceAI	MetaDome Analysis	Patient ID
*LAMA5* (NM_005560.6) 59 kb	c.7375G>A, p.(Ala2459Thr)	0.00003	D	D	T	D	NA	NA	Slightly intolerant	5
	c.2185G>A, p.(Gly729Ser)	2.2 × 10^−5^	D	D	D	D	NA	NA	Slightly intolerant	12
	c.1043C>T, p.(Ala348Val)	0.00004	D	D	D	D	NA	NA	Intolerant	23
	c.2248G>A, p.(Val750Met)	0.00011	D	D	D	D	NA	NA	Intolerant	55
	c.8269G>A, p.(Ala2757Thr)	4 × 10^−6^	D	D	D	D	NA	NA	Intolerant	77
*SYNE2* (NM_182914.3) 373 kb	c.12856A>C, p.(Ile4286Leu)	2.9 × 10^−5^	D	D	T	D	NA	NA	Neutral	16
	c.18001G>A, p.(Asp6001Asn)	0.00012	D	D	T	D	NA	NA	Intolerant	23
	c.15757G>T, p.(Glu5253 *)	NA	D	NA	NA	D	NA	NA	Tolerant	26
	c.16018G>T, p.(Val5340Phe)	2.8 × 10^−5^	D	D	D	D	NA	NA	Slightly tolerant	31
	c.18565C>T, p.(Arg6189Trp)	4 × 10^−6^	D	D	D	D	NA	NA	Slightly tolerant	45
*MAP3K4* (NM_005922.4) 126 kb	c.2566C>A, p.(Pro856Thr)	NA	D	D	T	D	NA	NA	Intolerant	4
	c.2659G>A, p.(Asp887Asn)	1.4 × 10^−5^	D	D	T	D	NA	NA	Intolerant	28, 63, 64 ¶
	c.3590_3598dupCTGCTGCTG, p.(Ala1197_Ala1199dup)	0.00014	D	NA	NA	NA	NA	NA	NA	68
*FCRL3* (NM_052939.4) 27 kb	c.1776_1783dupTCTGCTGC, p.(His595fs)	NA	D	NA	NA	NA	NA	NA	Neutral	2
	c.1643A>G, p.(Asn548Ser)	5.7 × 10^−5^	D	D	D	D	NA	NA	Intolerant	15
	c.958T>A, p.(Phe320Ile)	1.2 × 10^−5^	D	D	D	D	NA	NA	Tolerant	34, 35 ¶
*GREB1* (NM_014668.4) 109 kb	c.5780G>A, p.(Arg1927His)	3.2 × 10^−5^	D	D	D	D	NA	NA	Neutral	3
	c.5782G>A, p.(Asp1928Asn)	NA	D	D	D	D	NA	NA	Neutral	8
	c.1241C>T, p.(Ser414Phe)	2.8 × 10^−5^	D	D	T	D	NA	NA	Intolerant	54
*VEZT* (NM_017599.4) 85 kb	c.514T>C, p.(Trp172Arg)	0.00004	D	D	T	D	NA	NA	Intolerant	24
	c.1428G>T, p.(Lys476Asn)	0.00019	D	T	D	D	NA	NA	Intolerant	42, 61
*SYNE1* (NM_182961.4) 516 kb	c.21095A>G, p.(Gln7032Arg)	4.4 × 10^−5^	D	D	D	D	NA	NA	Intolerant	40
	c.16111C>T, p.(Arg5371 *)	1.6 × 10^−5^	D	NA	NA	D	NA	NA	Tolerant	47
*C3* (NM_000064.4) 53 kb	c.2951-5_2951-3delTGC	0.00074	NA	NA	NA	NA	NA	D	NA	24
	NM_000064.4:c.3431C>T	6.7 × 10^−5^	D	D	D	D	NA	NA	Intolerant	80
*MMP3* (NM_002422.5) 8.0 kb	c.1153G>A, p.(Val385Met)	0.00039	D	D	D	D	NA	NA	Intolerant	22
	c.484T>C, p.(Ser162Pro)	4 × 10^−6^	D	D	D	D	NA	NA	Neutral	32
*MMP9* (NM_004994.3) *7.7* kb	c.1420dupA, p.(Thr474fs)	0.00011	D	NA	NA	NA	NA	NA	Neutral	22
	c.1127C>T, p.(Thr376Ile)	4 × 10^−6^	D	D	T	D	NA	NA	Intolerant	36
*TYK2* (NM_003331.5) 30 kb	c.3475C>T, p.(Arg1159Cys)	1.2 × 10^−5^	D	D	D	D	NA	NA	Intolerant	15
	c.256C>A, p.(Pro86Thr)	5.3 × 10^−5^	D	D	D	D	NA	NA	Intolerant	59
*VEGFA* (NM_003376.6) 16 kb	c.337G>C, p.(Ala113Pro)	8 × 10^−6^	D	D	D	D	NA	NA	NA	67
	c.1184G>C, p.(Arg395Pro)	NA	D	D	D	D	NA	NA	Intolerant	70
*RHOJ* (NM_020663.4) 89 kb	c.554C>T, p.(Ala185Val)	2.5 × 10^−5^	D	T	D	D	NA	NA	Slightly tolerant	4, 69

**Table 3:** Private variants within single genes identified through WES analysis in individual EM patients. The table displays the rare, private, predicted, and damaging variants within single genes identified in individual patients of the EM cohort. All variants were detected at the heterozygous state. Gene name (isoform), size: name of the gene, isoform, and gene size. HGVS coding, protein: cDNA and protein change variant description according to the Human Genome Variation Society (HGVS) nomenclature guidelines. AF: gnomAD allele frequency. PaPI, PolyPhen, SIFT, DANN, dbscSNV, SpliceAI: variant effect evaluated via *in silico* prediction tools. MetaDome analysis: genetic-tolerance-profile domain of the identified variant. Patient ID: unique identifier of the patient carrying the variant. NA: not available. D: damaging. T: tolerated.

Gene Name (Isoform), Size	HGVS Coding, Protein	AF	PaPI	PolyPhen	SIFT	DANN	dbscSNV	SpliceAI	MetaDome Analysis	Patient ID
*KAZN* (NM_201628.2) 519 kb	c.236G>A, p.(Arg79Gln)	0.000049	D	D	T	D	NA	NA	Tolerant	3
*IL18* (NM_001562.3) 21 kb	c.113T>C, p.(Phe38Ser)	NA	D	D	D	D	NA	NA	Highly intolerant	9
*WT1* (NM_024426.6) 48 kb	c.475G>A, p.(Glu159Lys)	0.000034	D	T	D	D	NA	NA	NA	19
*CYP19A1* (NM_000103.4) 130 kb	c.1327G>A, p.(Ala443Thr)	0.000012	D	D	D	D	NA	NA	Neutral	50
*IL1A* (NM_000575.5) 11 kb	c.526G>C, p.(Asp176His)	0.000064	D	D	T	D	NA	NA	Slightly intolerant	67
*IL2RB* (NM_000878.3) 49 kb	c.1640C>G, p.(Pro547Arg)	NA	D	D	D	D	NA	NA	Slightly tolerant	14
*LILRB2* (NM_001278406.1) 7.4 kb	c.964C>T, p.(Arg322Cys)	0.000004	T	D	T	D	NA	NA	Slightly tolerant	14
*ZNF366* (NM_152625.1) 65 kb	c.1402G>A, p.(Val468Met)	NA	D	D	T	D	NA	NA	Intolerant	13

**Table 4:** Variants within novel genes identified through WES analysis in EM patients. The table displays the rare, predicted, and damaging variants within novel genes identified in patients of the EM cohort. All variants were detected at the heterozygous state. Gene name (isoform), size: name of the gene, isoform, and gene size. HGVS coding, protein: cDNA and protein change variant description according to the Human Genome Variation Society (HGVS) nomenclature guidelines. AF: gnomAD allele frequency. PaPI, PolyPhen, SIFT, DANN, dbscSNV, SpliceAI: variant effect evaluated via *in silico* prediction tools. MetaDome analysis: genetic-tolerance-profile domain of the identified variant. Patient ID: unique identifier of the patient carrying the identified variants. NA: not available. D: damaging. T: tolerated.

Gene Name (Isoform), Size	HGVS Coding, Protein	AF	PaPI	PolyPhen	SIFT	DANN	dbscSNV	SpliceAI	MetaDome Analysis	Patient ID
*ABCA13* (NM_152701.5) 476 kb	c.14579G>A, p.(Gly4860Glu)	0.000004	D	D	D	D	NA	NA	Intolerant	6
	c.2039A>G, p.(Asn680Ser)	0.000004	T	T	D	D	NA	NA	Slightly intolerant	9
	c.410_421delGACTTTGGGTAG, p.(Arg137_Glu141delinsLys)	0.00009	D	NA	NA	NA	NA	NA	NA	12
	c.13246A>G, p.(lle4416Val)	0.000007	T	D	T	D	NA	NA	Intolerant	14
	c.13243delC, p.(Ile4416fs)	0.000024	D	NA	NA	NA	NA	NA	Intolerant	27
	c.3248T>A, p.(Met1083Lys)	0.000032	D	T	D	D	NA	NA	Intolerant	31
	c.8030T>C, p.(Ile2677Thr)	NA	D	D	D	D	NA	NA	Highly tolerant	31
	c.11981C>T, p.(Ser3994Leu)	0.000024	D	D	D	D	NA	NA	Tolerant	56
	c.14185C>T, p.(Arg4729Cys)	0.000336	D	D	T	D	NA	NA	Slightly intolerant	75
*NEB* (NM_001164508.2) 249 kb	c.16817A>G, p.(Tyr5606Cys)	0.000098	D	D	D	D	NA	NA	Neutral	7
	c.8674C>T, p.(Leu2892Phe)	NA	D	D	T	D	NA	NA	Slightly intolerant	8
	c.4105G>A, p.(Glu1369Lys)	0.00002	D	D	T	D	NA	NA	Intolerant	13
	c.4558C>A, p.(Pro1520Thr)	NA	D	D	T	D	NA	NA	Intolerant	17
	c.4289T>A, p.(Ile1430Asn)	0.000004	D	D	D	D	NA	NA	Intolerant	32
	c.8317C>T, p.(Arg2773Trp)	0.000036	D	D	D	D	NA	NA	Neutral	33
	c.2771A>C, p.(Tyr924Ser)	0.000247	D	D	D	D	NA	NA	Intolerant	60
	c.18862G>A, p.(Val6288Ile)	0.000385	D	T	D	D	NA	NA	Tolerant	61
*CSMD1* (NM_033225.6) 2.1 Mb	c.2783C>T, p.(Ala928Val)	0.000004	D	D	T	D	D	NA	NA	10
	c.3023T>A, p.(Ile1008Asn)	NA	D	D	T	D	NA	NA	NA	17
	c.4553T>C, p.(Ile1518Thr)	0.000005	D	D	T	D	NA	NA	NA	58
	c.3333T>A, p.(Asn1111Lys)	0.000049	D	D	T	D	NA	NA	NA	62

## Data Availability

The genetic data described in this manuscript were submitted to the European Variation Archive and are accessible in variant call format at the following link: www.ebi.ac.uk/eva/?eva-study=PRJEB62099 (accessed on 9 May 2023).

## Supplementary Materials

The following supporting information can be downloaded at https://www.mdpi.com/article/10.3390/biomedicines11082122/s1, Table S1. List of the analysed genes via WES; Table S2. Complete WES analysis results of the EM patient cohort; Table S3. Burden of genes.
