# Supplementary material for: Puzzling Out the Genetic Architecture of Endometriosis: Whole-Exome Sequencing and Novel Candidate Gene Identification in a Deeply Clinically Characterised Cohort
Source: Biomedicines. 2023 Jul 27;11(8):2122. doi: 10.3390/biomedicines11082122 (PMC10452899; doi:10.3390/biomedicines11082122)
Supplement: Supplementary file 1 [file biomedicines-11-02122-s001.zip › Table S1.pdf]

Table S1. List of the analysed genes by WES. Gene: Gene name. Encoded protein: name of the encoded protein. Literature references: references and year of publication of the articles describing the gene in relation to EM. The novel candidate genes identified are reported in bold.

Gene	Encoded protein	Literature references
ABCA13	ATP-binding cassette transporter 13	NA
BDNF	Brain-Derived Neurotrophic Factor	doi: 10.1177/1933719117732161 (2018), doi: 10.1186/s13048-022-00963-9 (2022), doi: 10.1038/s41588-023-01323-z (2023)
C3	Complement C3	doi: 10.29271/jcpsp.2019.08.702 (2019), doi: 10.3389/fimmu.2021.693118 (2021)
CCDC170	Coiled-Coil Domain Containing 170	doi: 10.1038/ncomms15539 (2017), doi: 10.1093/molehr/gaaa082 (2021)
CEP112	Centrosomal Protein 112	doi: 10.1101/406967 (2018), https://doi.org/10.3892/wasj.2023.189 (2023)
COX2	Prostaglandin-Endoperoxide Synthase 2	https://doi.org/10.1186/2047-783X-17-12 (2012), doi: 10.7150/ijbs.35128 (2019), doi: 10.1016/j.tjog.2021.05.022 (2021)
CSMD1	CUB And Sushi Multiple Domains 1	NA
CYP19A1	Cytochrome P450 Family 19 Subfamily A Member 1	doi: 10.3390/ijms21218235 (2020), doi: 10.21873/invivo.11970 (2020), doi: 10.1016/j.ejogrb.2020.05.023 (2020)
ESR1	Estrogen Receptor 1	doi: 10.1038/ncomms15539 (2017), doi: 10.1093/molehr/gaaa082 (2021), doi: 10.1038/s41588-023-01323-z (2023)
FCRL3	Fc Receptor Like 3	doi: 10.1016/j.humimm.2011.05.005 (2011), doi: 10.1016/j.fertnstert.2012.01.125 (2012), doi: 10.1097/MD.0000000000001168 (2015)
FN1	Fibronectin 1	doi: 10.1136/jmedgenet-2012-101257 (2013), doi: 10.1038/ncomms15539 (2017), doi: 10.3892/mmr.2019.10247 (2019)
FSHB	Follicle Stimulating Hormone Subunit Beta	doi: 10.1038/ncomms15539 (2017), doi: 10.3389/fendo.2021.760616 (2021), doi: 10.1038/s41588-023-01323-z (2023)
GREB1	Growth Regulating Estrogen Receptor Binding 1	doi: 10.1017/thg.2015.61 (2015), doi: 10.1038/ncomms15539 (2017), doi: 10.3892/mmr.2019.10247 (2019), doi: 10.1038/s41588-023-01323-z (2023)
HOXA10	Homeobox A10	doi: 10.1177/1933719118768704 (2018), doi: 10.1186/s13104-018-3836-1 (2018), doi: 10.1177/1933719118766255 (2019), doi: 10.1038/s41588-023-01323-z (2023)
IL10	Interleukin 10	doi: 10.4103/1735-1995.166215 (2015), doi: 10.1038/cddis.2017.95 (2017), doi: 10.1093/humrep/deac248 (2023)
IL15	Interleukin 15	doi: 10.1530/REP-16-0089 (2016), doi: 10.1074/jbc.RA120.012753 (2020), doi: 10.1016/j.fertnstert.2021.11.024 (2022)

IL16	Interleukin 16	doi: 10.1111/iji.12281 (2016),<br>doi: 10.3892/ijmm.2018.3368 (2018),<br>https://doi.org/10.3892/wasj.2023.189 (2023)
IL17A	Interleukin 17A	doi: 10.3389/fimmu.2020.00108 (2020),<br>doi: 10.7150/ijms.71972 (2022),<br>doi: 10.1080/01443615.2023.2199852 (2023)
IL18	Interleukin 18	doi: 10.1111/j.1600-0897.2012.01147.x (2012),<br>doi: 10.1016/j.fertnstert.2016.07.005 (2016),<br>doi: 10.2174/0929866525666180412160045 (2018)
IL1A	Interleukin 1 alpha	doi: 10.1038/jhg.2013.32 (2013),<br>doi: 10.1093/humrep/deu267 (2015),<br>doi: 10.1080/09513590.2019.1631790 (2020)
IL1B	Interleukin 1 beta	doi: 10.1016/j.cyto.2015.12.015 (2017),<br>doi: 10.1093/molehr/gaz045 (2019),<br>doi: 10.1093/humrep/deaa017 (2020)
IL1R1	Interleukin 1 Receptor Type 1	doi: 10.1111/j.1600-0897.2012.01136.x (2012),<br>doi: 10.3389/fimmu.2019.02021 (2019)
IL2RB	Interleukin 2 Receptor Subunit Beta	doi: 10.1007/s43032-021-00751-8 (2022),<br>doi: 10.1186/s12916-022-02500-3 (2022)
IL4	Interleukin 4	doi: 10.1177/1933719115578930 (2015),<br>doi: 10.5114/ceji.2016.60992 (2016)
IL6	Interleukin 6	doi: 10.3892/etm.2017.4794 (2017),<br>doi: 10.1210/en.2017-00562 (2018),<br>doi: 10.1210/clinem/dgaa096 (2020),<br>doi: 10.1093/molehr/gaab064 (2021)
KAZN	Kazrin	doi: 10.1080/09513590.2018.1499090 (2019),<br>doi: 10.3892/etm.2019.7346 (2019)
KDR	Kinase Insert Domain Receptor	doi: 10.1159/000350665 (2013),<br>doi: 10.1038/ncomms12350 (2016),<br>doi: 10.1016/j.ejogrb.2016.10.046 (2017)
LAMA5	Laminin Subunit Alpha 5	doi: 10.1016/j.ejogrb.2013.10.007 (2013),<br>doi: 10.1080/09513590.2018.1499090 (2019)
LILRB1	Leukocyte Immunoglobulin Like Receptor B1	doi: 10.1007/s00438-017-1404-3 (2018),<br>doi: 10.3390/jcm8091468 (2019)
LILRB2	Leukocyte Immunoglobulin Like Receptor B2	doi: 10.1007/s00438-017-1404-3 (2018),<br>https://www.spandidos-publications.com/10.3892/wasj.2023.189 (2023)
MAP3K4	Mitogen-Activated Protein Kinase Kinase Kinase 4	doi: 10.1093/humrep/dex024 (2017),<br>doi: 10.1111/jog.14710 (2021),<br>https://doi.org/10.3892/wasj.2023.189 (2023)
MMP2	Matrix Metallopeptidase 2	doi: 10.5604/12321966.1226861 (2016),<br>doi: 10.1371/journal.pone.0163540 (2016)
MMP3	Matrix Metallopeptidase 3	doi: 10.1002/mrd.22931 (2018),<br>doi: 10.1016/j.eurox.2019.100041 (2019),<br>doi: 10.3390/ijms21082840 (2020)
MMP9	Matrix Metallopeptidase 9	doi: 10.5604/12321966.1226861 (2016),<br>doi: 10.3390/diagnostics10060364 (2020)

NEB	Nebulin	NA
NGF	Nerve Growth Factor	doi: 10.1016/j.jogoh.2020.101895 (2021),<br>doi: 10.1038/s41588-023-01323-z (2023)
RHOA	Ras Homolog Family Member A	doi: 10.4103/1673-5374.170325 (2015),<br>doi: 10.1111/jcmm.15689 (2020)
RHOJ	Ras Homolog Family Member J	doi: 10.4103/1673-5374.170325 (2015),<br>doi: 10.1016/j.ejogrb.2017.08.037 (2017)
RND3	Rho Family GTPase 3	doi: 10.1002/cphy.c150018 (2015),<br>doi: 10.4103/1673-5374.170325 (2015),<br>doi: 10.3389/fmolb.2021.743012 (2021)
SKAP1	Src Kinase Associated Phosphoprotein 1	doi: 10.1016/j.xhgg.2023.100185 (2023),<br>doi: 10.1038/s41588-023-01323-z (2023)
SYNE1	Spectrin Repeat Containing Nuclear Envelope Protein 1	doi: 10.1038/ncomms15539 (2017),<br>doi: 10.1038/s41588-023-01323-z (2023)
SYNE2	Spectrin Repeat Containing Nuclear Envelope Protein 2	doi: 10.1210/en.2014-1490 (2014),<br>doi: 10.3390/cells10040749 (2021)
TNF	Tumor Necrosis Factor	doi: 10.1177/1933719114536472 (2015),<br>doi: 10.1111/aji.13305 (2020)
TYK2	Tyrosine Kinase 2	doi: 10.1016/j.humimm.2011.12.009 (2012),<br>doi: 10.1016/j.humimm.2012.09.007 (2013)
VEGF	Vascular Endothelial Growth Factor A	doi: 10.1016/j.fertnstert.2015.12.016 (2016),<br>doi: 10.1007/s11033-019-04807-6. (2019)
VEZT	Vezatin	doi: 10.1093/humrep/dev022 (2015),<br>doi: 10.1093/humrep/dew047 (2016),<br>doi: 10.3390/ijms21051765 (2017)
WNT4	Wnt Family Member 4	doi: 10.1007/s10815-015-0523-1 (2015),<br>doi: 10.3892/mmr.2017.7398 (2017)
WT1	WT1 Transcription Factor	doi: 10.1038/s41588-023-01323-z (2023),<br>https://doi.org/10.3390/reprodmed1010003 (2020)
ZNF366	Zinc Finger Protein 366	doi: 10.1155/2015/461024 (2015),<br>doi: 10.3892/etm.2019.7346 (2019)
