# Supplementary material for: Puzzling Out the Genetic Architecture of Endometriosis: Whole-Exome Sequencing and Novel Candidate Gene Identification in a Deeply Clinically Characterised Cohort
Source: Biomedicines. 2023 Jul 27;11(8):2122. doi: 10.3390/biomedicines11082122 (PMC10452899; doi:10.3390/biomedicines11082122)
Supplement: Supplementary file 1 [file biomedicines-11-02122-s001.zip › Table S2.pdf]

Table S2. Complete WES analysis results of the EM patient cohort. The table reports, for each patient, the rare damaging variants within the selected list of genes and the novel candidates identified through WES analyses. Patient ID: patient unique identifier. Gene: gene name. HGVS coding and protein: cDNA and protein change variant description according to the Human Genome Variation Society (HGVS) nomenclature guidelines. AF: gnomAD allele frequency. PaPI, PolyPhen, SIFT, DANN, dbscSNV, SpliceAI: variant effect evaluated by the in-silico prediction tools. *: stop codon. NA: not available. D: damaging. T: tolerated. In bold are reported the novel candidate genes.

Patient ID	Gene	HGVS coding	HGVS protein	AF	dbSNP ID	PaPI	PolyPhen	SIFT	DANN	dbsc-SNV	Splice-AI
1	–	–	–	–	–	–	–	–	–	–	–
2	*FCRL3*	NM_052939.3:c.1776_1783dupTCTGCTGC	p.(His595fs)	NA	NA	D	NA	NA	NA	NA	NA
3	*KAZN*	NM_201628.2:c.236G>A	p.(Arg79Gln)	0.000049	rs754907438	D	D	T	D	NA	NA
	GREB1	NM_014668.3:c.5780G>A	p.(Arg1927His)	0.000032	s376907059	D	D	D	D	NA	NA
4	*RHOJ*	NM_020663.4:c.554C>T	p.(Ala185Val)	0.000025	rs145036225	D	T	D	D	NA	NA
	MAP3K4	NM_005922.2:c.2566C>A	p.(Pro856Thr)	NA	NA	D	D	T	D	NA	NA
5	*LAMA5*	NM_005560.4:c.7375G>A	p.(Ala2459Thr)	0.000030	rs774378028	D	D	T	D	NA	NA
6	***ABCA13***	NM_152701.3:c.14579G>A	p.(Gly4860Glu)	0.000004	rs1343617695	D	D	D	D	NA	NA
7	***NEB***	NM_001164508.1:c.16817A>G	p.(Tyr5606Cys)	0.000098	rs372049328	D	D	D	D	NA	NA
8	*GREB1*	NM_014668.3:c.5782G>A	p.(Asp1928Asn)	NA	NA	D	D	D	D	NA	NA
	NEB	NM_001164508.1:c.8674C>T	p.(Leu2892Phe)	NA	NA	D	D	T	D	NA	NA
9	***ABCA13***	NM_152701.3:c.2039A>G	p.(Asn680Ser)	0.000004	rs1213044373	T	T	D	D	NA	NA
	IL18	NM_001562.3:c.113T>C	p.(Phe38Ser)	NA	NA	D	D	D	D	NA	NA
10	***CSMD1***	NM_033225.5:c.2783C>T	p.(Ala928Val)	0.000004	rs1486469825	D	D	T	D	D	NA
11	–	–	–	–	–	–	–	–		–	–
12	***ABCA13***	NM_152701.3:c.410_421delGACTTTGGGTAG	p.(Arg137_Glu141delinsLys)	0.000090	rs775345564	D	NA	NA	NA	NA	NA
	LAMA5	NM_005560.4:c.2185G>A	p.(Gly729Ser)	0.000022	rs758295582	D	D	D	D	NA	NA

13	***NEB***	NM_001164508.1:c.4105G>A	p.(Glu1369Lys)	0.000020	rs779213215	D	D	T	D	NA	NA
	ZNF366	NM_152625.1:c.1402G>A	p.(Val468Met)	NA	NA	D	D	T	D	NA	NA
14	*IL2RB*	NM_000878.3:c.1640C>G	p.(Pro547Arg)	NA	NA	D	D	D	D	NA	NA
	ABCA13	NM_152701.5:c.13246A>G	p.(Ile4416Val)	0.000007	rs761788799	T	D	T	D	NA	NA
	LILRB2	NM_001278406.1:c.964C>T	p.(Arg322Cys)	0.000004	rs768452192	T	D	T	D	NA	NA
15	*TYK2*	NM_003331.4:c.3475C>T	p.(Arg1159Cys)	0.000012	rs753470142	D	D	D	D	NA	NA
	FCRL3	NM_052939.3:c.1643A>G	p.(Asn548Ser)	0.000057	rs140292392	D	D	D	D	NA	NA
16	*SYNE2*	NM_182914.2:c.12856A>C	p.(Ile4286Leu)	0.000029	rs763081757	D	D	T	D	NA	NA
17	***NEB***	NM_001164508.1:c.4558C>A	p.(Pro1520Thr)	NA	NA	D	D	T	D	NA	NA
	CSMD1	NM_033225.5:c.3023T>A	p.(Ile1008Asn)	NA	NA	D	D	T	D	NA	NA
18	—	—	—	—	—	—	—	—	—	—	—
19	*WT1*	NM_024426.6:c.475G>A	p.(Glu159Lys)	0.000034	rs768165877	D	T	D	D	NA	NA
20	—	—	—	—	—	—	—	—	—	—	—
21	—	—	—	—	—	—	—	—	—	—	—
22	*MMP9*	NM_004994.3:c.1420dupA	p.(Thr474fs)	0.000107	rs765108149	D	NA	NA	NA	NA	NA
	MMP3	NM_002422.5:c.1153G>A	p.(Val385Met)	0.000393	rs202217886	D	D	D	D	NA	NA
23	*SYNE2*	NM_182914.3:c.18001G>A	p.(Asp6001Asn)	0.000119	rs769737846	D	D	T	D	NA	NA
	LAMA5	NM_005560.6:c.1043C>T	p.(Ala348Val)	0.000040	rs201517990	D	D	D	D	NA	NA
24	*VEZT*	NM_017599.4:c.514T>C	p.(Trp172Arg)	0.000040	rs757379531	D	D	T	D	NA	NA
	C3	NM_000064.4:c.2951-5_2951-3delTGC	NA	0.000742	rs544122376	NA	NA	NA	NA	NA	D
25	—	—	—	—	—	—	—	—	—	—	—
26	*SYNE2*	NM_182914.3:c.15757G>T	p.(Glu5253*)	NA	NA	D	NA	NA	D	NA	NA
27	***ABCA13***	NM_152701.5:c.13243delC	p.(Ile4416fs)	0.000024	rs761151004	D	NA	NA	NA	NA	NA

28	*MAP3K4*	NM_005922.4:c.2659G>A	p.(Asp887Asn)	0.000014	rs776867715	D	D	T	D	NA	NA
29	—	—	—	—	—	—	—	—	—	—	—
30	—	—	—	—	—	—	—	—	—	—	—
31	***ABCA13***	NM_152701.5:c.3248T>A	p.(Met1083Lys)	0.000032	rs918676610	D	T	D	D	NA	NA
	ABCA13	NM_152701.5:c.8030T>C	p.(Ile2677Thr)	NA	rs778285795	D	D	D	D	NA	NA
	SYNE2	NM_182914.3:c.16018G>T	p.(Val5340Phe)	0.000028	rs200214648	D	D	D	D	NA	NA
32	***NEB***	NM_001164508.2:c.4289T>A	p.(Ile1430Asn)	0.000004	rs773404854	D	D	D	D	NA	NA
	MMP3	NM_002422.5:c.484T>C	p.(Ser162Pro)	0.000004	NA	D	D	D	D	NA	NA
33	***NEB***	NM_001164508.2:c.8317C>T	p.(Arg2773Trp)	0.000036	rs776175154	D	D	D	D	NA	NA
34	*FCRL3*	NM_052939.4:c.958T>A	p.(Phe320Ile)	0.000012	rs754875256	D	D	D	D	NA	NA
35	*FCRL3*	NM_052939.4:c.958T>A	p.(Phe320Ile)	0.000012	rs754875256	D	D	D	D	NA	NA
36	*MMP9*	NM_004994.3:c.1127C>T	p.(Thr376Ile)	0.000004	rs777794038	D	D	T	D	NA	NA
37	—	—	—	—	—	—	—	—	—	—	—
38	—	—	—	—	—	—	—	—	—	—	—
39	—	—	—	—	—	—	—	—	—	—	—
40	*SYNE1*	NM_182961.4:c.21095A>G	p.(Gln7032Arg)	0.000044	rs752508132	D	D	D	D	NA	NA
41	—	—	—	—	—	—	—	—	—	—	—
42	*VEZT*	NM_017599.4:c.1428G>T	p.(Lys476Asn)	0.000185	rs200452809	D	T	D	D	NA	NA
43	—	—	—	—	—	—	—	—	—	—	—
44	—	—	—	—	—	—	—	—	—	—	—
45	*SYNE2*	NM_182914.3:c.18565C>T	p.(Arg6189Trp)	0.000004	rs1366404251	D	D	D	D	NA	NA
46	—	—	—	—	—	—	—	—	—	—	—
47	*SYNE1*	NM_182961.4:c.16111C>T	p.(Arg5371*)	0.000016	rs772587027	D	NA	NA	D	NA	NA

48	—	—	—	—	—	—	—	—	—	—	—
49	—	—	—	—	—	—	—	—	—	—	—
50	*CYP19A1*	NM_000103.4:c.1327G>A	p.(Ala443Thr)	0.000012	rs201638381	D	D	D	D	NA	NA
51	—	—	—	—	—	—	—	—	—	—	—
52	—	—	—	—	—	—	—	—	—	—	—
53	—	—	—	—	—	—	—	—	—	—	—
54	*GREB1*	NM_014668.4:c.1241C>T	p.(Ser414Phe)	0.000028	rs747899314	D	D	T	D	NA	NA
55	*LAMA5*	NM_005560.6:c.2248G>A	p.(Val750Met)	0.000107	rs201119098	D	D	D	D	NA	NA
56	***ABCA13***	NM_152701.5:c.11981C>T	p.(Ser3994Leu)	0.000024	rs371333701	D	D	D	D	NA	NA
57	—	—	—	—	—	—	—	—	—	—	—
58	***CSMD1***	NM_033225.6:c.4553T>C	p.(Ile1518Thr)	0.000005	rs756141932	D	D	T	D	NA	NA
59	*TYK2*	NM_003331.5:c.256C>A	p.(Pro86Thr)	0.000053	rs141466711	D	D	D	D	NA	NA
60	***NEB***	NM_001164508.2:c.2771A>C	p.(Tyr924Ser)	0.000247	rs199903114	D	D	D	D	NA	NA
61	***NEB***	NM_001164508.2:c.18862G>A	p.(Val6288Ile)	0.000385	rs201886728	D	T	D	D	NA	NA
	VEZT	NM_017599.4:c.1428G>T	p.(Lys476Asn)	0.000185	rs200452809	D	T	D	D	NA	NA
62	***CSMD1***	NM_033225.6:c.3333T>A	p.(Asn1111Lys)	0.000049	rs371414165	D	D	T	D	NA	NA
63	*MAP3K4*	NM_005922.4:c.2659G>A	p.(Asp887Asn)	0.000014	rs776867715	D	D	T	D	NA	NA
64	*MAP3K4*	NM_005922.4:c.2659G>A	p.(Asp887Asn)	0.000014	rs776867715	D	D	T	D	NA	NA
65	—	—	—	—	—	—	—	—	—	—	—
66	—	—	—	—	—	—	—	—	—	—	—
67	*VEGFA*	NM_003376.6:c.337G>C	p.(Ala113Pro)	0.000008	rs915247074	D	D	D	D	NA	NA
	IL1A	NM_000575.5:c.526G>C	p.(Asp176His)	0.000064	rs1801715	D	D	T	D	NA	NA
68	*MAP3K4*	NM_005922.4:c.3590_3598dupCTGCTGCTG	p.(Ala1197_Ala1199dup)	0.000136	rs763893932	D	NA	NA	NA	NA	NA

69	*RHOJ*	NM_020663.4:c.554C>T	p.(Ala185Val)	0.000025	rs145036225	D	T	D	D	NA	NA
70	*VEGFA*	NM_003376.6:c.1184G>C	p.(Arg395Pro)	NA	NA	D	D	D	D	NA	NA
71	—	—	—	—	—	—	—	—	—	—	—
72	—	—	—	—	—	—	—	—	—	—	—
73	—	—	—	—	—	—	—	—	—	—	—
74	—	—	—	—	—	—	—	—	—	—	—
75	***ABCA13***	NM_152701.5:c.14185C>T	p.(Arg4729Cys)	0.000336	rs185678311	D	D	T	D	NA	NA
76	—	—	—	—	—	—	—	—	—	—	—
77	*LAMA5*	NM_005560.6:c.8269G>A	p.(Ala2757Thr)	0.000004	rs762279213	D	D	D	D	NA	NA
78	—	—	—	—	—	—	—	—	—		—
79	—	—	—	—	—	—	—	—	—	—	—
80	*C3*	NM_000064.4:c.3431C>T	p.(Thr1144Met)	0.000067	rs150237828	D	D	D	D	NA	NA
