# Supplementary material for: Puzzling Out the Genetic Architecture of Endometriosis: Whole-Exome Sequencing and Novel Candidate Gene Identification in a Deeply Clinically Characterised Cohort
Source: Biomedicines. 2023 Jul 27;11(8):2122. doi: 10.3390/biomedicines11082122 (PMC10452899; doi:10.3390/biomedicines11082122)
Supplement: Supplementary file 1 [file biomedicines-11-02122-s001.zip › Table S3.pdf]

Table S3. Burden of genes. The table shows, for each analyzed individual in the EM cohort and in the control group, the "burden of genes", defined as the total number of genes in which at least a rare predicted damaging variant was identified, after WES analysis and variants selection**.** Cohort: name of the cohort to which each analysed individual belongs. Sample ID: individual unique identifier. Burden of genes: for each individual, the total number of genes within the analysed genes in which at least a rare predicted damaging variant was identified, after WES analysis and variants selection.

Cohort	Sample ID	Burden of genes
EM	1	0
EM	2	1
EM	3	2
EM	4	2
EM	5	1
EM	6	1
EM	7	1
EM	8	2
EM	9	2
EM	10	1
EM	11	0
EM	12	2
EM	13	2
EM	14	3
EM	15	2
EM	16	1
EM	17	2
EM	18	0
EM	19	1
EM	20	0
EM	21	0
EM	22	2
EM	23	2
EM	24	2
EM	25	0
EM	26	1
EM	27	1
EM	28	1
EM	29	0
EM	30	0
EM	31	2
EM	32	2
EM	33	1
EM	34	1
EM	35	1
EM	36	1
EM	37	0

EM	38	0
EM	39	0
EM	40	1
EM	41	0
EM	42	1
EM	43	0
EM	44	0
EM	45	1
EM	46	0
EM	47	1
EM	48	0
EM	49	0
EM	50	1
EM	51	0
EM	52	0
EM	53	0
EM	54	1
EM	55	1
EM	56	1
EM	57	0
EM	58	1
EM	59	1
EM	60	1
EM	61	2
EM	62	1
EM	63	1
EM	64	1
EM	65	0
EM	66	0
EM	67	2
EM	68	1
EM	69	1
EM	70	1
EM	71	0
EM	72	0
EM	73	0
EM	74	0
EM	75	1
EM	76	0
EM	77	1
EM	78	0
EM	79	0
EM	80	1

Controls	1C	0
Controls	2C	0
Controls	3C	1
Controls	4C	0
Controls	5C	0
Controls	6C	0
Controls	7C	1
Controls	8C	1
Controls	9C	1
Controls	10C	1
Controls	11C	0
Controls	12C	0
Controls	13C	1
Controls	14C	2
Controls	15C	1
Controls	16C	1
Controls	17C	1
Controls	18C	0
Controls	19C	0
Controls	20C	0
Controls	21C	1
Controls	22C	0
Controls	23C	1
Controls	24C	1
Controls	25C	0
Controls	26C	0
Controls	27C	1
Controls	28C	1
Controls	29C	0
Controls	30C	1
Controls	31C	0
Controls	32C	0
Controls	33C	1
Controls	34C	1
Controls	35C	1
Controls	36C	1
Controls	37C	1
Controls	38C	2
Controls	39C	0
Controls	40C	0
Controls	41C	1
Controls	42C	1
Controls	43C	1

Controls	44C	0
Controls	45C	0
Controls	46C	0
Controls	47C	0
Controls	48C	1
Controls	49C	1
Controls	50C	0
Controls	51C	0
Controls	52C	0
Controls	53C	1
Controls	54C	3
Controls	55C	0
Controls	56C	0
Controls	57C	0
Controls	58C	0
Controls	59C	1
Controls	60C	1
Controls	61C	2
Controls	62C	1
Controls	63C	2
Controls	64C	1
Controls	65C	1
Controls	66C	1
Controls	67C	1
Controls	68C	0
Controls	69C	2
Controls	70C	0
Controls	71C	0
Controls	72C	0
Controls	73C	2
Controls	74C	0
Controls	75C	1
Controls	76C	1
Controls	77C	1
Controls	78C	0
Controls	79C	2
Controls	80C	0
Controls	81C	0
Controls	82C	1
Controls	83C	2
Controls	84C	1
Controls	85C	2
Controls	86C	1

Controls	87C	0
Controls	88C	0
Controls	89C	0
Controls	90C	0
Controls	91C	0
Controls	92C	0
Controls	93C	0
Controls	94C	0
Controls	95C	2
Controls	96C	0
Controls	97C	1
Controls	98C	1
Controls	99C	0
Controls	100C	0
Controls	101C	0
Controls	102C	0
Controls	103C	0
Controls	104C	0
Controls	105C	0
